# The correlation between blood-lipid ratio in the first trimester and large-for-gestational-age infants

**DOI:** 10.1186/s12944-023-01781-8

**Published:** 2023-02-01

**Authors:** Zixuan Wang, Yaru Peng, Shufang Mao, Liqian Zhang, Yanwei Guo

**Affiliations:** 1 Department of Obstetrics and Gynecology, The Affiliated Hospital of Chengde Medical University, Chengde, 067000 PR China; 2 Department of Preventive Medicine, Chengde Medical University, Chengde, 067000 PR China; 3 Department of Gynaecology, Chengde Medical University, Chengde, 067000 PR China

**Keywords:** First trimester, Glucose/lipid metabolism, Blood lipid ratio, Large-for-gestational-age infants, Predictive value

## Abstract

**Background:**

To investigate the correlation between maternal glucose and lipid metabolism indexes and blood-lipid ratio in the first trimester and large-for- gestational-age (LGA) infants.

**Methods:**

Women in the first trimester of pregnancy who underwent regular obstetric examination in the obstetric outpatient department of the Affiliated Hospital of Chengde Medical College from June 2018 to March 2019 were included according to the standard. Basic information were collected based on questionnaires at the first visit of pregnant women, including early fasting blood glucose (FBG), fasting insulin (FINS), glycated hemoglobin (HbA1c), high-density lipoprotein (HDL), low-density lipoprotein (LDL), triglyceride (TG), total cholesterol (TC), apolipoprotein A1 (APO-A1), apolipoprotein B (APO-B), lipoprotein a (LP(a)), LDL/HDL, TG/HDL, TC/HDL, APO-B/APO-A1 ratio, birth weight of newborns, gestational age at delivery etc.

**Results:**

A total of 418 cases were included for analysis. The incidence rate of LGA infants was 13.88%, and that of small-for-gestational-age (SGA) infants was 4.78%. Univariate analysis revealed that the age, pre-pregnancy body mass index (BMI), weight gain during pregnancy, APO-B/APO-A1 between LGA group and appropriate-for-gestational-age (AGA) group were significantly different (*P* < 0.05); multivariate stepwise logistic regression analysis indicated that the correlation between maternal age, pre-pregnancy BMI, weight gain during pregnancy, APO-B/APO-A1 level and LGA were statistically significant (*P* < 0.05); compared with the reference range of APO-B/APO-A1 of 0.46–0.65, values < 0.46 and > 0.65 were protective factor of LGA (*P* < 0.05). The receiver operating curve(ROC) indicated that the area under the curve (AUC)s for predicting LGA using maternal age, pre-pregnancy BMI, weight gain during pregnancy, and early pregnancy APO-B/APO-A1 were 0.585, 0.606, 0.637, 0.531, respectively. The AUC for a combined prediction model was 0.742, showing greater predictive value than any other factors individually.

**Conclusion:**

Maternal age, pre-pregnancy BMI, weight gain during pregnancy, and APO-B/APO-A1 levels in first trimester are significant factors influencing the occurrence of LGA infants, and the combination of the four factors would have certain predictive value for LGA.

## Background

During pregnancy, the mother undergoes many adaptive changes to meet the needs of fetal growth and development, and change in glucose and lipid metabolism is one of the main changes [1, 2]. Although abnormal glucose and lipid metabolism and neonatal birth weight in the second and third trimesters of pregnancy have been relatively extensively studied, the measures aiming to control fetal weight by regulating the level of blood sugar and blood lipids in the second and third trimesters are limited due to the special period of pregnancy [3–8]. This study focuses on the relationship between glucose and lipid metabolism levels, blood/lipid ratios and Large for-gestational-age (LGA) in first trimester. LGA generally implies a birth weight equal to or more than the 90th percentile for a given gestational age [9]. In addition to the routinely used indicators, low-density lipoprotein/high-density lipoprotein (LDL/HDL), triglyceride /high-density lipoprotein( TG/HDL), total Cholesterol/high-density lipoprotein /(TC/HDL), apolipoprotein B / apolipoprotein A1 (APO-B/APO-A1) ratio were included in the analysis. An indicator closely related to the birth weight of the newborn in the first trimester was expected to provide a more reliable theoretical basis for the supervision and management of pregnancy and early intervention, in order to reduce the incidence of adverse neonatal outcomes.

### Objects and methods

#### Research objects

Women in the first trimester who underwent regular obstetric examination at the obstetric outpatient department of the Affiliated Hospital of Chengde Medical College from June 2018 to March 2019 and planned to give birth in this hospital were included. All patients included in the study were informed, expressed their willingness to participate, and signed an informed consent form at the first check-up at 6–14 weeks of pregnancy. The approval of the Medical Ethics Committee of this hospital was obtained. Inclusion criteria: permanent residents of Chengde city (2 consecutive years or more), aged 18–45 years, naturally conceived, singleton, gestational age 6–14 weeks, and planned to deliver in this hospital. Exclusion criteria: suffering from type 1 or type 2 diabetes mellitus, chronic hypertension, dyslipidemia detected before pregnancy, cardiovascular disease, etc.; acute or chronic liver, kidney or other organ diseases; smoking and/or drinking history; recent use of drugs that affect glucose and lipid metabolism. This study initially included 510 women in first trimester, and 6 cases with missing laboratory indicators and 11 cases with fetal abortion or fetal malformation were excluded. Another 75 pregnant women who did not deliver in our hospital as planned were excluded due to incomplete information, and there were 418 pregnant women with complete information.

## Methods

### Data collection

The heights of the pregnant women were measured during the first prenatal visit at 6–14 weeks of pregnancy. The basic information was collected through questionnaires at the first visit of pregnant women, including the demographic characteristics (age, ethnicity, place of permanent residence, education level, per capita household income), last menstrual period, maternal history, family history, previous medical history (pre-pregnancy diabetes, cardiovascular and cerebrovascular diseases, dyslipidemia), drug application history, smoking and alcohol history, body weight one month prior to pregnancy. Body mass index (BMI) is a statistical index using a person's weight and height to provide an estimate of body fat in males and females of any age. BMI = weight (kg)/ [height (m)]^2^. When the pre-pregnancy BMI is below 18.5, the normal weight gain during-pregnancy is 12.5 to 18 kg. When the BMI is between 18.5–24.9, a normal weight is 7–11.5 kg; in a similar sense, when the BMI is greater than or equal to 30, a normal weight gain is considered to be 5–9 kg. When the during-pregnancy weight gain of a patient is below the weight ranges, it was ruled as insufficient weight gain while a weight gain is above the ranges, it was ruled as excessive [10]. Other information of pregnant women who met the inclusion criteria in the first trimester were collected through the hospital digital information management system, including fasting blood glucose (FBG), fasting insulin (FINS), glycated hemoglobin (HbA1c), high-density lipoprotein (HDL), low-density lipoprotein (LDL), triglyceride (TG), total Cholesterol (TC), apolipoprotein A1 (APO-A1), apolipoprotein B (APO-B), lipoprotein a (LP(a)), LDL/HDL, TG/HDL, TC/HDL, APO-B/APO-A1 ratios, and neonatal birth weight, gestational age at delivery, etc. Collection and detection of glucose and lipid metabolism indicators: All included subjects were 6–14 weeks pregnant. They were fasted for 8–12 h overnight, and 5 ml of cubital vein blood was drawn at 8:00–9:00 the next morning with coagulant. After setting at room temperature for 30 min, samples were centrifuge at 2,500 rpm for 15 min, and serum were taken for test. Among them, FBG, HbA1c, HDL, LDL, TG, TC, APO-A1, APO-B, LP(a), LDL/HDL, TG/HDL, TC/HDL, APO-B/APO-A1 were analyzed using 5600 automatic biochemical immunoanalyzer. FINS were determined using double-antibody sandwich immunoelectrochemiluminescence; FBG, HbA1c, TG and TC were determined by hexokinase method, ion-exchange high-performance liquid chromatography, GPO-POD method and enzymatic method, respectively; HDL and LDL were read by direct method; LP(a), APO-A1, and APO-B were determined by immunoturbidimetric endpoint method.

### Statistical analysis

SPSS 26.0 software was used for statistical analysis. In this study, all data tested using a Kolmogorv-Smirnov test. the P values are all below 0.05 indicates that data did not follow a normal distribution and the P value greater than 0.05 indicates that data followed a normal distribution. Measurement data subject to normal distribution is expressed as mean ± standard deviation. Independent-samples t test is used for comparison between two groups. For measurement data that does not obey normal distribution is described by M (P25-P75), independent-samples Wilcoxon sank sum test was used for comparison between two groups. Enumeration data were described by composition ratio or rate, and comparison between groups was done by chi-square test or Fisher's exact probability method. Multivariate stepwise logistic regression analysis was performed on the influencing factors of LGA (α_in_ = 0.05, α _out_ = 0.10). Using ROC to analyze the predictive value of each indicators for LGA. The significant level for other hypothesis tests is 0.05.

## Results

This study initially included 510 women in first trimester, and 6 cases with missing laboratory indicators and 11 cases with fetal abortion or fetal malformation were excluded. Another 75 pregnant women who did not deliver in our hospital as planned were excluded due to incomplete information, and there were 418 pregnant women with complete information. According to gestational age and birth weight of newborns, they were divided into three groups: AGA, LGA, and SGA. All 418 cases were included in the final analysis.

### Basic characteristics of subjects and birth weight of newborns

Among the 418 pregnant women included in the analysis, the age ranged from 20 to 44 years, with an average age of 29.31 ± 4.24 years. The average pre-pregnancy BMI was 22.71 ± 3.77 kg/m^2^, pre-pregnancy underweight pregnant women accounted for 12.2% (51/418), overweight pregnant women accounted for 25.5% (107/418), and pre-pregnancy obese pregnant women accounted for 9.02% (39/418). Excessive and insufficient weight gain during pregnancy accounted for 59.33% (248/418) and 10.53% (44/418), respectively; while normal weight gain during pregnancy accounted for 30.14% (126/418). The incidence of LGA was 13.88% (58/418), and that of SGA was 4.78% (20/418).

### Comparison of the basic characteristics and the levels of blood glucose and blood lipids of pregnant women in the first trimester between LGA and AGA groups 

The age, pre-pregnancy BMI and weight gain of pregnant women in the LGA group were higher than those in the AGA group, with significant differences (*P* < 0.05). Other factors including FBG, FINS, HbA1c, HDL, LDL, TG, TC, APO-A1, APO-B, LP(a), LDL/HDL, TG/HDL, TC/HDL, APO-B/APO-A1 levels in the first trimester were comparable between the two groups (*P* > 0.05, Tables 1 and 2).

**Table 1:** Comparison of the basic characteristics of pregnant women in the LGA group and the AGA group and the levels of blood glucose and blood lipids in the first trimester [M (P25-P75), $\overline x$ ± s]

Group	Cases	Age (y)	Pre-pregnancy BMI	Gestational age at delivery (w)	Weight gain during pregnancy (kg)	FBG (mmol/L)	FINS (uIU/mL)	HbA1c (%)
AGA group	340	29.18 ± 4.06	22.70 ± 3.90	39.17 ± 1.42	16.02 ± 5.02	4.75 ± 0.71	63.18(44.98 ~ 93.22)	4.9 ± 0.61
LGAgroup	58	30.48 ± 4.43	23.90 ± 3.53	39.31 ± 1.06	18.38 ± 5.06	4.78 ± 0.41	72.98(53.43 ~ 92.48)	4.8 ± 0.31
t/z		2.223	2.194	0.726	3.304	0.282	1.594	0.503
P		0.027	0.029	0.468	0.001	0.278	0.111	0.615

**Table 2:** .

Group	HDL (mmol/L)	LDL (mmol/L)	TG (mmol/L)	TC (mmol/L)	APO-A1 (g/L)	APO-B (g/L)	LP(a) (mg/L)	LDL/HDL	TG/HDL	TC/HDL	APO-B/APO-A1
AGA group	1.60 ± 0.32	1.9 ± 0.54	1.32(1.01 ~ 1.70)	4.20 ± 0.74	1.42 ± 0.22	0.79 ± 0.20	10.30(4.80 ~ 23.10)	1.24 ± 0.41	0.96 ± 0.55	2.69 ± 0.55	0.57 ± 0.16
LGA group	1.65 ± 0.39	1.9 ± 0.45	1.42(1.10 ~ 2.03)	4.25 ± 0.76	1.46 ± 0.27	0.81 ± 0.17	10.55(4.68 ~ 24.40)	1.21 ± 0.33	1.10 ± 0.74	2.64 ± 0.46	0.57 ± 0.13
t/z	1.023	0.025	1.646	0.486	1.046	0.715	0.319	0.566	1.358	0.597	0.097
P	0.307	0.98	0.100	0.627	0.296	0.475	0.749	0.572	0.179	0.551	0.923

### Comparison of glucose and lipid metabolism indexes and blood lipid ratio in LGA group and AGA group in the first trimester

The glucose and lipid metabolism indicators of the 418 pregnant women included in the analysis were arranged in order from small to large, and the values at first quartile, third quartile. The values are shown in the table below. They were divided into three groups < P25, P25-P75, > P75 with P25 and P75 as cut-off points, and the differences in the composition of LGA and AGA among different levels of glucose and lipid metabolism indicators in the first trimester were compared. The results showed that there was a statistically significant difference in the composition of LGA and AGA with different APO-B/APO-A1 levels in the first trimester (*P* < 0.05) (Tables 3 and 4).

**Table 3:** Comparison of glucose and lipid metabolism indexes and composition of blood lipid ratio in the first trimester between LGA and AGA groups [n (%)]

	FBG (mmol/L)	FINS (uIU/mL)	HbA1c (%)	HDL (mmol/L)	LDL (mmol/L)	TG (mmol/L)	TC (mmol/L)	APO-A1 (g/L)	APO-B (g/L)	LP(a) (mg/L)	LDL/HDL	TG/HDL	TC/HDL	APO-B/APO-A1
P_25_	4.51	46.01	4.70	1.38	1.55	1.02	3.71	1.28	0.67	4.80	0.96	0.62	2.29	0.46
P_75_	4.94	92.32	5.10	1.82	2.23	1.72	4.64	1.57	0.90	23.70	1.46	1.12	3.04	0.65

**Table 4:** .

Characteristics	AGA group	LGA group	χ^2^	*P*	Characteristics	AGA group	LGA group	χ^2^	*P*
	(*n* = 340)	(*n* = 58)				(*n* = 340)	(*n* = 58)		
FBG(mmol/L)			5.434	0.067	APO-A1(g/L)			1.571	0.439
< 4.51	89 (26.2)	13 (22.4)			< 1.28	95 (28.1)	12 (20.7)		
4.51–4.94	171 (50.3)	23 (39.7)			1.28–1.57	161 (47.6)	29 (50.0)		
> 4.94	80 (23.5)	22 (37.9)			> 1.57	82 (24.3)	17 (29.3)		
FINS(uIU/mL)			2.551	0.284	APO-B(g/L)			1.557	0.454
< 46.01	89 (26.2)	10 (17.2)			< 0.67	94 (27.9)	13 (22.4)		
46.01–92.32	166 (48.8)	34 (58.6)			0.67–0.90	157 (46.6)	26 (44.8)		
> 92.32	85 (25.0)	14 (24.1)			> 0.90	86 (25.5)	19 (32.8)		
HbA1c(%)			0.375	0.839	LP(a)(mg/L)			0.063	0.967
< 4.70	104 (30.6) (30.6)	17 (29.3)			< 4.80	89 (26.4)	15 (25.9)		
4.70–5.10	138 (40.6)	22 (37.9)			4.80–23.70	166 (49.3)	28 (48.3)		
> 5.10	98 (28.8)	19 (32.8)			> 23.70	82 (24.3)	15 (25.9)		
HDL(mmol/L)			0.285	0.862	LDL/HDL			5.011	0.086
< 1.38	89 (26.2)	15 (25.9)			< 0.96	91 (26.8)	11 (19.0)		
1.38–1.82	168 (49.4)	27 (46.6)			0.96–1.46	157 (46.2)	36 (62.1)		
> 1.82	83 (24.4)	16 (27.6)			> 1.46	92 (27.1)	11 (19.0)		
LDL(mmol/L)			1.041	0.598	TG/HDL			2.189	0.356
< 1.55	96 (28.2)	13 (22.4)			< 0.62	86 (25.3)	13 (22.4)		
1.55–2.23	160 (47.1)	31 (53.4)			0.62–1.12	163 (47.9)	24 (41.4)		
> 2.23	84 (24.7)	14 (24.1)			> 1.12	91 (26.8)	1 (36.2)		
TG(mmol/L)			4.745	0.090	TC/HDL			1.936	0.391
< 1.02	90 (26.5)	10 (17.2)			< 2.29	91 (26.8)	13 (22.4)		
1.02–1.72	169 (49.7)	27 (46.6)			2.29–3.04	160 (47.1)	33 (56.9)		
> 1.72	81 (23.8)	21 (36.2)			> 3.04	89 (26.2)	12 (20.7)		
TC(mmol/L)			1.677	0.442	APO-B/APO-A1			10.392	0.007
< 3.71	88 (25.9)	16 (27.6)			< 0.46	93 (27.6)	9 (15.5)		
3.71–4.64	175 (51.5)	25 (43.1)			0.46–0.65	144 (42.7)	38 (65.5)		
> 4.64	77 (22.6)	17 (29.3)			> 0.65	100 (29.7)	11 (19.0)		

### Multivariate logistic regression analysis of the relationship between glucose and lipid metabolism indexes, blood lipid ratio and LGA in the first trimester

The LGA stepwise logistic regression analysis was performed by taking LGA as the dependent variable (LGA = 1, AGA = 0), and the variables with *P* < 0.1 in the above univariate analysis (age, pre-pregnancy BMI, weight gain during pregnancy, FBG, TG, LGL/HDL, APO-B/APO-A1) as the independent variable. Given that it is believed in the clinical work that the older the gestational age at delivery results in the longer growth of the fetus in the womb and greater the birth weight, the gestational age at delivery is also used as an independent variable. As shown in the Tables 5 and 6 the maternal age, pre-pregnancy BMI, weight gain during pregnancy, APO-B/APO-A1 level were significantly correlated with LGA (*P* < 0.05); compared with APO-B/APO-A1 in the reference range of 0.46–0.65, both < 0.46 and > 0.65 were protective factors for LGA (*P* < 0.05).

**Table 5:** Logistic regression analysis of the relationship between glucose and lipid metabolism indexes, blood lipid ratio and LGA in the first trimester

Variables	Assignment
APO-B/APO-A1 < 0.46	1
0.46 < APO-B/APO-A1 < 0.65	2
APO-B/APO-A1 > 0.65	3

**Table 6:** .

Variables	Β value	SE value	Wald value	*P* value	aOR value	95% CL	
						Lower limit	Upper limit
Constant	-8.484	1.604	27.964	0.000	0.000		
Age	0.08	0.035	5.346	0.021	1.083	1.012	1.159
pre-pregnancy BMI	0.118	0.04	8.682	0.003	1.126	1.04	1.218
weight gain during pregnancy	0.123	0.032	14.446	0.000	1.131	1.061	1.204
APO-B/APO-A1			11.408	0.003			
2					1		
1	-1.101	0.412	7.153	0.007	0.332	0.148	0.745
3	-1.026	0.388	6.985	0.008	0.358	0.168	0.767

**Fig. 1:**
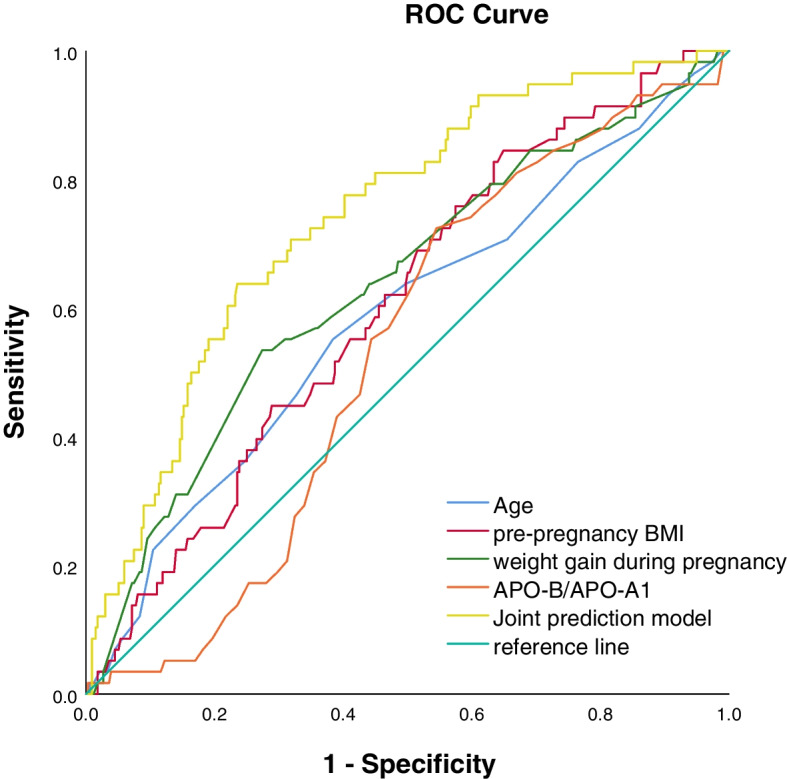
ROC curve of maternal age, pre-pregnancy BMI, gestational weight gain, APO-B/APO-A1 and the combination the four in predicting LGA

According to the logistic regression analysis, the ROC curves of maternal age, pre-pregnancy BMI, gestational weight gain, APO-B/APO-A1, and the combined prediction model of the four were drawn, as shown in Fig. 1, and the area under the curve (AUC) was calculated. The results show that the AUC of the joint prediction model is 0.742, which is more accurate than the single detection of maternal age, pre-pregnancy BMI, pregnancy weight gain, and APO-B/APO-A1 (AUCs are 0.585, 0.606, 0.637, and 0.531, respectively), and the difference was statistically significant (*P* < 0.05). Pre-pregnancy BMI has the highest sensitivity but the lowest specificity, APO-B/APO-A1 has high sensitivity but low specificity, and the sensitivity and specificity of the joint prediction model are relatively high (Table 7).

**Table 7:** The application value of maternal age, pre-pregnancy BMI, gestational weight gain, APO-B/APO-A1 and the combination of the four in predicting LGA

Variable(s)	AUC	Sensitivity	Specificity
Age^a^	0.585	55.20	61.60
pre-pregnancy BMI^a^	0.606	84.50	35.10
weight gain during pregnancy^a^	0.637	53.40	72.60
APO-B/APO-A1^a^	0.531	72.40	45.50
Joint prediction model	0.742	63.80	76.50

Joint prediction model:Age + pre-pregnancy BMI + weight gain during pregnancy + APO-B/APO-A1 Joint prediction

^a^Compared to the AUC of Joint prediction model, the difference was statistically significant (*P* < 0.05)

## Discussion

High neonatal birth weight leads to increased short-term complications for fetuses, newborns and pregnant women [11]; but in the long run, metabolic diseases such as obesity and type 2 diabetes may also occur, with greatly elevated risk of cardiovascular disease [12]. The theory of fetal origin of adult diseases [13] believes that a series of effects on the fetus during the intrauterine period have a profound impact on the physical condition of the same child and adult. Therefore, it is of particular importance to identify indicators that are closely related to LGA in the first trimester to guide the supervision and management of pregnancy.

The study revealed that the LGA group of pregnant women was older than the AGA pregnant women, and the probability of giving birth to LGA was 1.012 to 1.159 times the original level for every 1-year increase in age of pregnant women, which was consistent with the findings from Schimmel MS [14]. However, in the study by Li Sisi et al. [15], the incidence of low birth weight infants the highest in the two age groups of ≤ 24 years old and ≥ 40 years old. This suggests that with the increase of age, especially after the age of 40, women's physical function gradually declines, entering the menopausal transition period, then insufficient reserved ovarian function and disturbance of the endocrine environment may no longer provide a suitable growth environment for the fetus, which in turn affects the growth of the fetus. Therefore, in order to avoid the adverse effects of high or low birth weight of newborns, it is imperative to give birth at an appropriate age.

In this study, pre-pregnancy BMI and weight gain during pregnancy were correlated with LGA. Although pregnant women’s pre-pregnancy weight are all from their memories, which may be biased, several studies have confirmed that the self-reported height and weight of pregnant women are highly consistent with the actual value, which can be used to assess the actual situation of pre-pregnancy BMI [16, 17].

For every 1 kg/m^2^ increase in pre-pregnancy BMI, the risk of LGA increased by 12.6% compared with the original level; for every 1 kg of weight gain during pregnancy, the risk of LGA was 1.131 times higher. This is consistent with the findings by Feng Yinhong [18], Wang Fen [19] and others. Excessive accumulation of fat will reduce insulin sensitivity, leading to secondary hyperinsulinemia and eventually insulin resistance; in addition, hyperinsulinemia will in turn promote fat synthesis, slow down fat decomposition, and aggravate obesity. Both pre-pregnancy overweight or obesity, or obesity caused by excess weight gain during pregnancy, enable the occurrence of gestational diabetes mellitus(GDM) and LGA.

### Comparisons with similar studies and the implications of current work

In recent years, with the rapid progress in blood lipid research, it has been suggested that the predictive value of blood lipid ratios for insulin resistance, diabetes, metabolic syndrome, atherosclerosis, etc. is significantly better than that of a single blood lipid index [20, 21]. In a study of 1597 pregnant women by Zhao Yaping [22], the LDL/HDL and TG/HDL ratios of GDM pregnant women were significantly higher than those of non-GDM pregnant women, and the LDL/HDL and TG/HDL ratios in the second trimester had certain predictive value for macrosomia. Zhang Fanfan et al. [23] have found that the APO-B/APO-A1 level of GDM pregnant women was greater than that of non-GDM pregnant women, and APO-B/APO-A1 combined with HOMA-IR had a high predictive value for adverse pregnancy outcomes such as macrosomia.

In addition, the level of APO-B/APO-A1 in the first trimester was closely related to LGA and was an independent influencing factor of LGA. Compared with the level of 0.46–0.65, APO-B/APO-A1 < 0.46 and > 0.65 were both protective factor for LGA. Lipoproteins are composed of lipid and protein, and the protein part called apolipoprotein (APO), is responsible for lipid transport. Common lipoproteins include LDL, HDL, etc. LDL transports TC in the liver to the outside, resulting in hypertriglyceridemia, and TC deposits on the blood vessel wall, causing damage to the vascular endothelium. APO-B is responsible for transporting LDL; HDL transports extrahepatic TC to the liver for metabolism to reduce the TC in peripheral blood. APO-A1 mainly exists in HDL, so APO-B/APO-A1 can reflect the levels of LDL and HDL to a certain extent. It has been reported that [21] lipid-lowering drugs such as statins have little effect on APO-A1 and APO-B, thus APO-A1 and APO-B can better reflect the lipid metabolism in the body. When APO-B/APO-A1 is at a low level (APO-B/APO-A1 < 0.46), LDL and HDL metabolism are relatively normal, so the risk of LGA is lower than that of 0.46–0.65; when APO-B/APO-A1 is > 0.65, indicative of abnormal levels of LDL and HDL, hypertriglyceridemia will cause damage vascular endothelial cells regardless of high LDL or low HDL. When the uteroplacental vascular endothelium is damaged, insufficient nutritional supply of the placenta will later hinder the growth and development of the fetus. Hence, when the level of APO-B/APO-A1 > 0.65, the risk of delivery of LGA is reduced instead.

With the increasing research on lipids during pregnancy, it has been affirmed that maternal lipid metabolism is closely related to fetal development. At present, it is believed that the levels of triglyceride and cholesterol rise in the 9th to 13th week of pregnancy. The timing of blood collection in this study is from 6 to 14th week of pregnancy, which is in the initial stage of blood lipid changes. Therefore, in this study, the APO-B/ The performance of APO-A1 alone in predicting LGA is not competent (AUC = 0.531), but maternal age, pre-pregnancy BMI, gestational weight gain combined with APO-B/APO-A1 in the first trimester have a relatively high value in predicting the occurrence of LGA (AUC = 0.742), and the predictive performance is significantly better than that of a single variable. The ability of pregnant women to adjust the physiological changes of pregnancy varies, which suggests that the management of women in the first trimester of pregnancy should be strengthened, especially women with high risk factors in the first trimester of pregnancy.

### Strength and limitations

#### Strengths

In this study, multiple factors that are potentially related to LGA were analyzed; after screening of various mixed factors, it has been demonstrated that early pregnancy APO/APO-A1 ratio, age, pre-pregnancy BMI and during-pregnancy weight gain have significant correlation with LGA. By constructing ROC and calculating the area below the curve, it has been shown that the combination of the four factors has a greater predictive value for LGA than individual factors.

### Limitations

First, this study is conducted in only one medical institution, with relatively small sample size and potential bias. Future results would be more convincing if multiple institutions in the region can be combined to formulate a multi-center study. Second, this study is a prospective study, and the participants are pregnant women of 6–14 weeks involving a large range of gestational weeks. While expanding the sample size in the future, the grouping can be further refined to clarify the changes in blood lipids in different stages of early pregnancy, and to provide a powerful reference for formulating diagnostic criteria for hyperlipidemia in pregnancy. Third, this study focuses on the relationship between blood lipid ratio and LGA only in the first trimester, and its predictive value is limited. Longitudinal research can be combined with the results of the second and third trimesters to improve the predictive efficiency.

## Conclusions

As a result, APO-B/APO-A1 < 0.46 is a protective factor for LGA, however, when APO-B/APO-A1 > 0.65 is reached, possible vascular endothelial injury may hinder the normal growth and development of the fetus. In conclusion, APO-B/APO-A1 in the first trimester is a vital factor for the LGA outcome, and the management of blood lipids in this period should be carefully monitored to avoid adverse pregnancy outcomes. From the analysis of APO-B/APO-A1 ratio in the first trimester of pregnancy, along with ages of pregnant patients, pre-pregnancy BMI and weight gains during pregnancy, it was found that the combination of four has a certain predictive value for the occurrence of LGA. During regular pregnancy check-ups, practitioners should be encouraged to administrate dynamic monitoring to patients in their first trimesters regarding the during-pregnancy weight gains, while improving their APO-B/APO-A1 ratio. Obstetricians should provide guidance concerning dietary, nutritional and exercise-wise choices to pregnant patients with risk factors, which may bring positive influence on preventing from LGA occurrence.

## Data Availability

Dataset may be available, please contact corresponding author.

## Abbreviations

GDM: Gestational diabetes mellitus
LGA: Large-for-gestational age
FBG: Fasting blood glucose
FINS: Fasting insulin
HbA1c: Glycated hemoglobin
HDL: High-density lipoprotein
LDL: Low-density lipoprotein
TG: Triglyceride
TC: Total cholesterol
APO-A1: Apolipoprotein A1
APO-B: ApolipoproteinB
LP(a): Lipoprotein a
SGA: Small-for-gestational-age
AGA: Appropriate-for-gestational-age
BMI: Body mass index
ROC: Receiver operating curve
AUC: Area under the curve
